# Isolated Splenic Metastasis From Large-Cell Neuroendocrine Carcinoma of Lung: A Case Report

**DOI:** 10.7759/cureus.67491

**Published:** 2024-08-22

**Authors:** Hsuan Hung Yu, Ming Sung Yang, Ming Hsun Yang

**Affiliations:** 1 Department of Surgery, Cheng Hsin General Hospital, Taipei, TWN

**Keywords:** large-cell neuroendocrine carcinoma, splenectomy, non-small cell lung cancer, neuroendocrine carcinoma, isolated splenic metastasis

## Abstract

Splenic malignancies are mostly primary and lymphocytic. Metastases to the spleen are rare and imply tumor dissemination. Limited cases were reporting isolated splenic metastasis from non-small cell cancer of the lung (NSCLC). We report the case of a 68-year-old male with mixed large-cell neuroendocrine carcinoma (LCNEC) and adenocarcinoma of the lung who presented with asymptomatic, synchronous, and isolated splenic metastasis. The patient refused adjuvant or neoadjuvant therapies. Surgical removal of both primary and metastatic lesions was achieved separately. In the scenario of isolated splenic metastasis, local consolidative therapy such as splenectomy appears to benefit survival by alleviating tumor burden. The patient is currently disease-free after one year of postoperative follow-up.

## Introduction

Non-small cell lung cancer (NSCLC) represents approximately 85% of all lung cancers [1]. Major histological subtypes of NSCLC consist of adenocarcinoma, squamous cell carcinoma, and large cell carcinoma. Splenic metastases from solid tumors are uncommon. Previous reported origins of splenic metastases include breast cancer, lung cancer, colorectal cancer, ovarian cancer, and melanoma [2]. The reported prevalence of splenic metastasis from all solid tumors ranges from 2.3 to 7.1% [3]. Furthermore, isolated splenic metastasis from large-cell neuroendocrine carcinoma (LCNEC) is extremely rare. LCNEC of the lung is a highly aggressive tumor with neuroendocrine differentiation and neuroendocrine marker expression. Clinical features of LCNEC include old age, male predominance, and heavy smoking [4]. Treatment protocols for advanced-stage LCNEC are not yet established. Patients may be treated with SCLC-like or NSCLC-like chemotherapy [5]. We herein present a case of lung-mixed LCNEC and adenocarcinoma with isolated splenic metastasis.

## Case presentation

**Figure 1:**
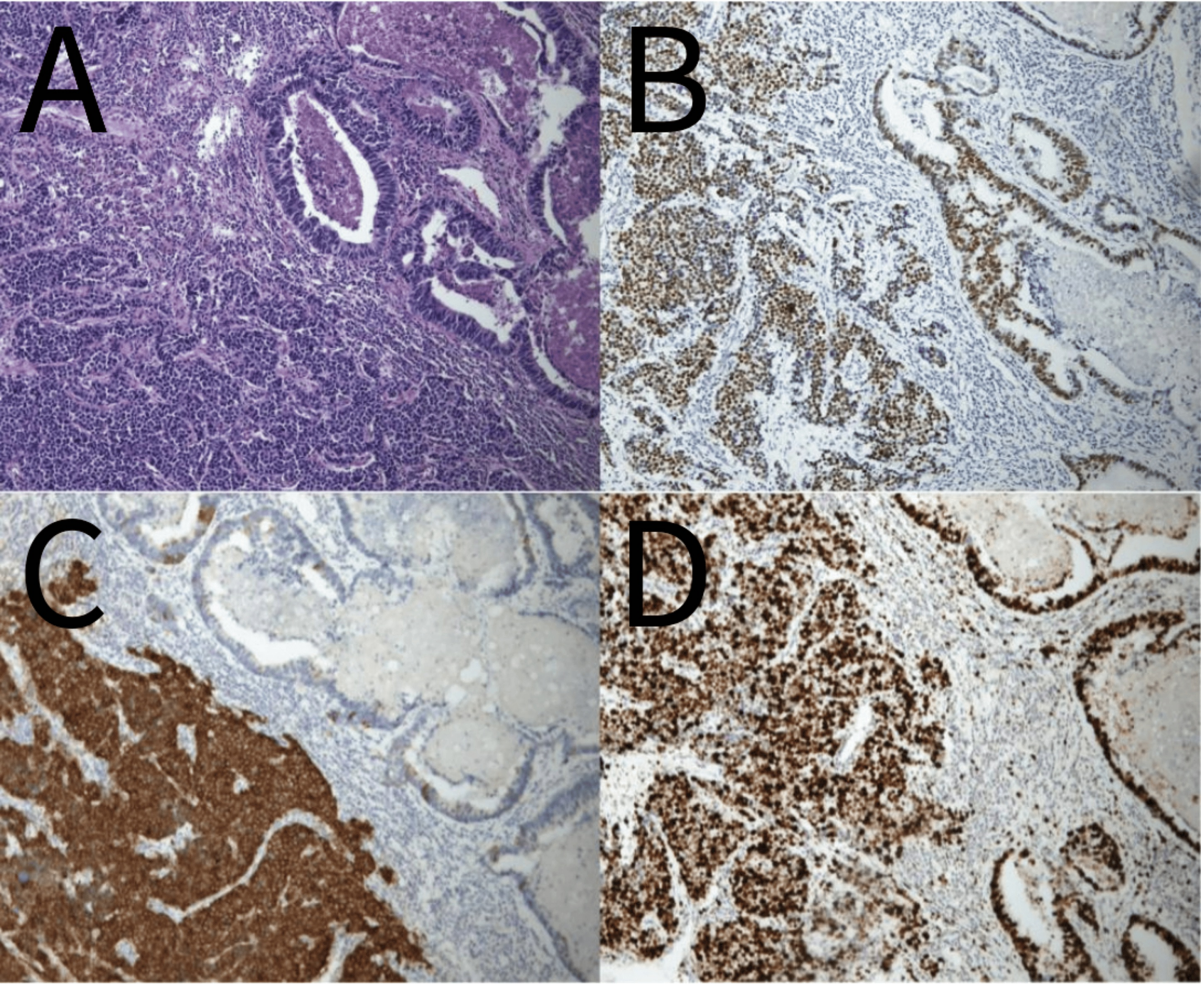
Microscopic view of the resected lung tumor. (A) Hematoxylin and eosin staining (magnification, x100) of the resected lung. Immunohistochemical stain demonstrated diffusely reactive to (B) TTF-1, (C) synaptophysin, and (D) Ki-67 (magnification, x100).

A 68-year-old, ex-smoking male with a medical history of hypertension and diabetes presented to the outpatient clinic due to unintentional weight loss of over 10 kilograms for three months. Chest X-ray revealed a 6.6 x 5.1 cm mass at the right upper lobe of the lung. Chest computed tomography (CT) was arranged, and soft tissue masses were disclosed at the right upper lung and spleen. CT-guided lung biopsy was performed and pathology reported non-small-cell lung cancer with positive neuroendocrine markers. Abdominal CT unveiled several variable-sized, heterogeneous, contrast-enhanced low-density masses in the enlarged spleen with a diameter of 20 centimeters. Whole-body positron emission tomography demonstrated tumor avidity in the right upper lung and enlarged spleen, consistent with primary splenic metastasis (Figure 1). 

**Figure 2:**
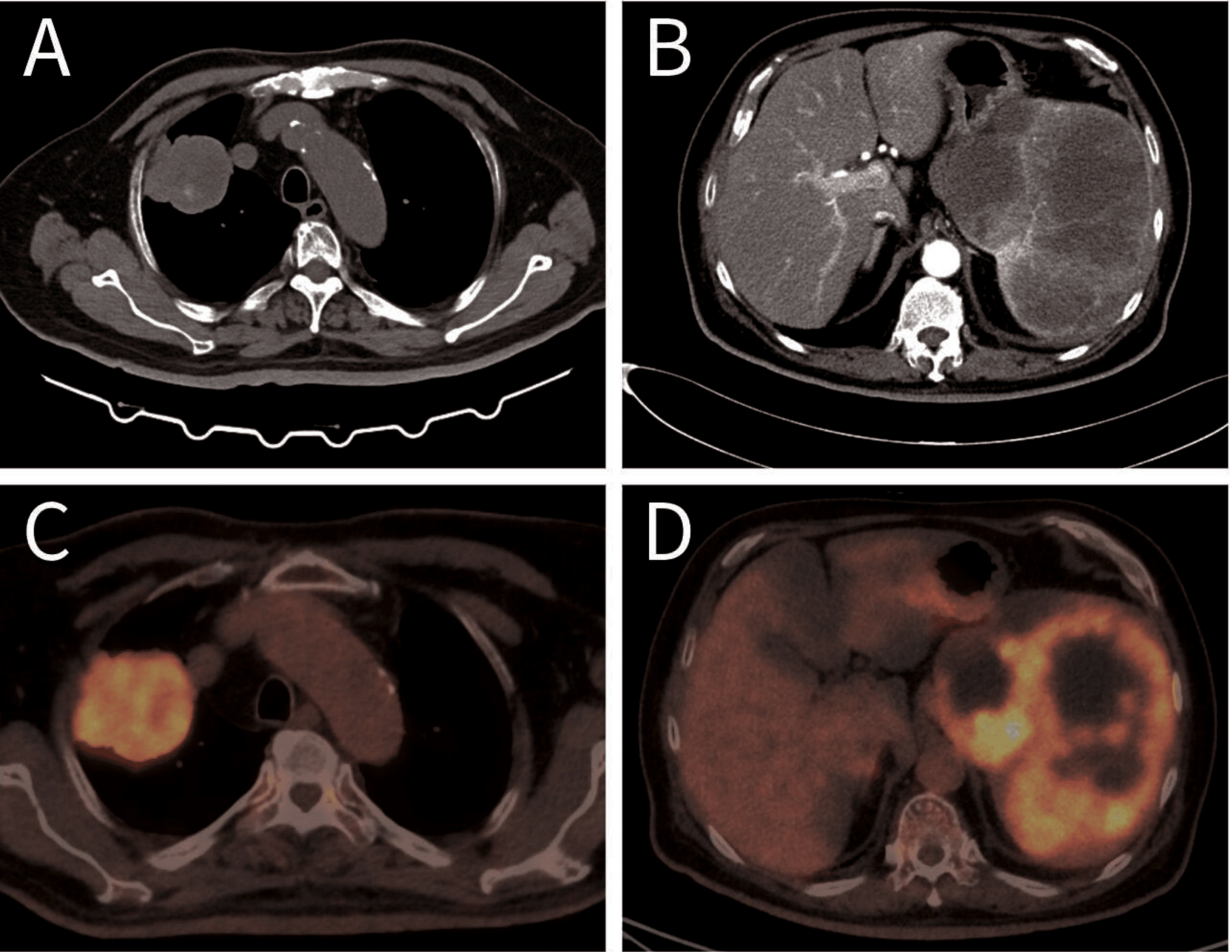
Preoperative axial image of CT and FDG/PET. (A) Soft tissue mass at right upper lung. (B) Several variable-sized heterogeneously contrast-enhanced relatively low-density masses are seen in the enlarged spleen. (C) 18F-FDG-PET/CT image showed (C) FDG-avid mass in the right upper lung, and (D) spleen with central absence of 18F-FDG avidity. FDG, fluorodeoxyglucose.

The tumor was staged cT3N1M1c. Interval removal of both tumors was planned. Laparoscopic procedures seemed to be impossible because of tumor size. The patient underwent open splenectomy and partial resection of the diaphragm. The resected spleen weighed a 1300 grams and the tumor was 17 x 13 x 9 centimeters in size. The cut surface was yellowish-white and multifocal nodularity was noticed. Microscopically, the tumor cells are diffusely reactive to TTF-1, synaptophysin, and CD56, compatible with metastatic large-cell neuroendocrine carcinoma (Figure 2). 

**Figure 3:**
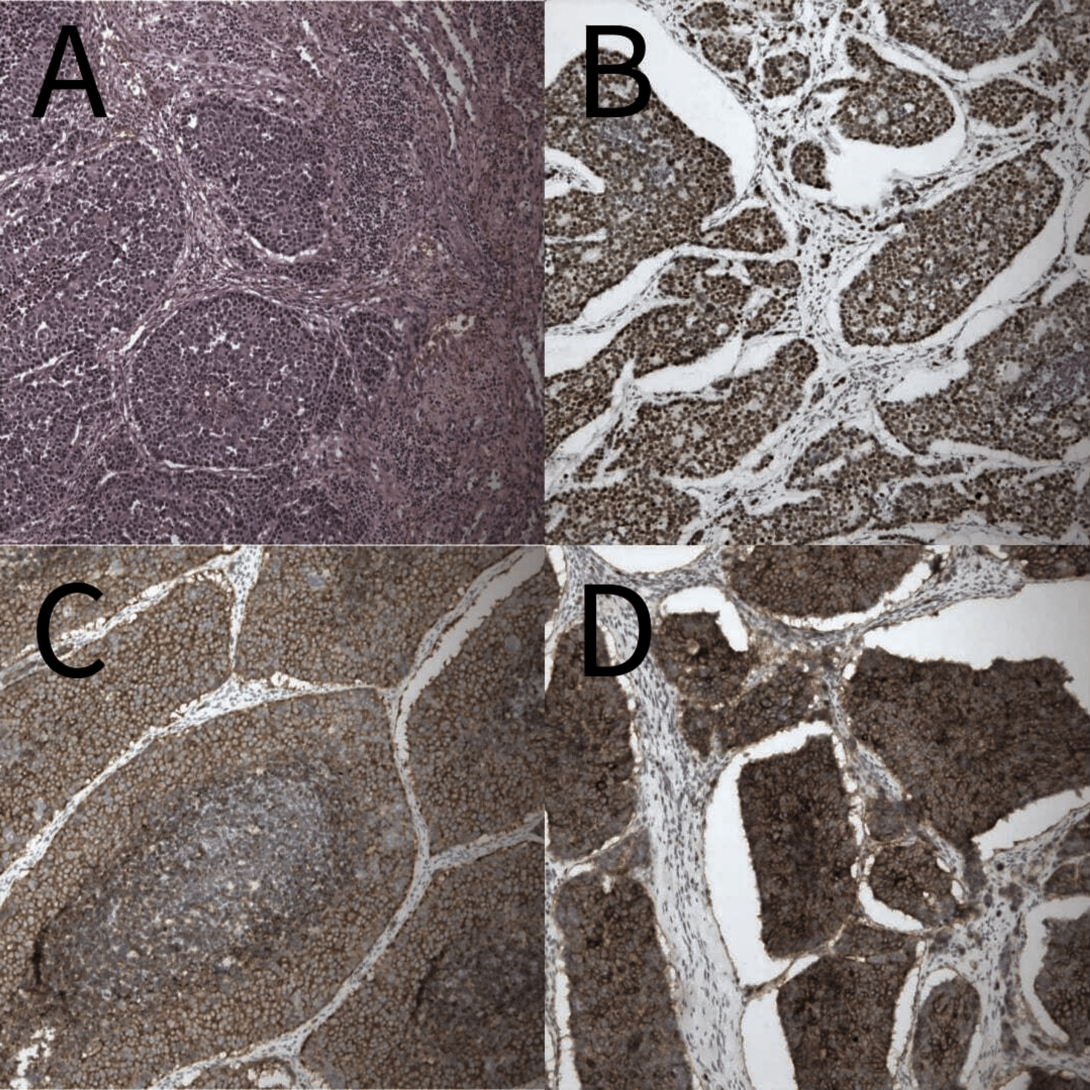
Microscopic view of the resected splenic tumor. The resected splenic tumor resembled the resected lung tumor. (A) Hematoxylin and eosin staining (magnification, x100) of the resected spleen, the tumor is composed of round tumor cells. Immunohistochemical stain demonstrated diffusely reactive to (B) TTF-1, (C) synaptophysin, and (D) CD56 (magnification, x100).

Surgical removal of the primary tumor was performed two months later. Pathology reported mixed adenocarcinoma, moderately differentiated, and large-cell neuroendocrine carcinoma (Figure 3), with R0 resection and negative regional lymph node metastasis.

Thus the final stage was pT3N0M1c, stage IVB. The patient refused neoadjuvant or adjuvant systemic therapies. There is no evidence of recurrence after one year of postoperative follow-up.

## Discussion

Herein we presented a case of right upper lung mixed adenocarcinoma and large-cell neuroendocrine carcinoma with isolated splenic metastasis. We found 37 cases of isolated metastasis from primary lung cancer in the medical literature. This could be the first case reporting splenic metastasis from large-cell neuroendocrine carcinoma.

Isolated splenic metastasis occurs almost equally regardless of the laterality of the primary lesion. Most cases with isolated splenic metastasis were diagnosed synchronously. On the other hand, it could be diagnosed metachronously after primary lung tumor resection. The longest interval was reported in a 56-year-old male in whom splenic metastasis was detected 144 months after surgery for left lung adenosquamous carcinoma [6].

The reported incidence of splenic metastasis in patients with primary lung cancer is between 1.6% and 5.6% [7]. Most secondary splenic lesions are seen in the terminal stage [2]. The liver, bone, adrenal gland, and central nervous system are the common sites for metastatic non-small-cell lung cancer [8]. In a retrospective study reviewing patients with uncommon metastases from non-small cell lung cancer, the incidence of splenic metastasis is reported to be 0.66% [9]. Several reasons can contribute to the rarity of splenic metastasis. The constant blood flow and the sharp angle between the splenic artery and the celiac axis, contraction of the splenic capsule, and the antiblastic activity of the spleen have been suggested to prevent tumor cell implantation [10-12].

Most patients were asymptomatic while some patients presented with abdominal pain and fever [3]. Notably, there are some cases presented with spontaneous, non-traumatic splenic rupture. Treatment modalities in such clinical emergencies included trans-arterial splenic embolization and urgent splenectomy [11-14]. One case was successfully managed with trans-arterial embolization [12].

Splenic metastases can be identified with abdominal sonography or CT as part of examinations in initial staging or surveillance. Common diagnostic features of malignant splenic lesions in CT are the absence of splenomegaly, ill-defined margin, absence of wall, solid nature, and absence of calcification [15,16]. However, polycystic change can also occur [17]. Calcification is unusual unless the primary tumor is mucinous adenocarcinoma [16,18]. The splenic metastases were detected in some case reports utilizing 18F-fluorodeoxyglucose (FDG)-PET/CT [6,14,19]. Nevertheless, the absence of 18F-FDG uptake can be observed when mucus is produced by the metastatic tumor [16,17].

Patients with metastatic lung cancer have been treated with chemotherapy, immunotherapy, or local consolidative therapy and palliative care to improve quality of life and relieve symptoms. Oligometastasis is a concept that has been defined as cancer with no more than five metastatic sites and three organs. Current NCCN (National Comprehensive Cancer Network) guidelines recommend definitive local therapies for oligometastases including but not limited to the brain and adrenal gland. Moreover, according to ACCP (American College of Chest Physicians) guidelines, if patients have synchronous, resectable N0, primary NSCLC with isolated brain or adrenal gland metastasis, resection of metastases is recommended [20]. However, surgical resection for splenic or other distant metastases is not well-discussed in the guidelines due to the scarcity of patient entities. One study enrolled 37 patients with isolated adrenal metastasis from NSCLC who underwent adrenalectomy or non-operative treatment [21]. The authors concluded that surgical resection provided a better median survival rate (19 vs. 6 months, p=0.005) and five-year survival (34% vs. 0%, p=0.002). Prognostic factors included the presence of mediastinal nodal disease and a lesion contralateral to its origin.

Neoadjuvant or adjuvant chemotherapy is rational in stage IV lung cancer but the patient refused any systemic therapy. Despite the unintentional weight loss, the patient demonstrated a good performance status (ECOG 0), therefore surgery was indicated. There were case reports mentioning laparoscopic or hand-assisted splenectomy for metastatic splenic lesions [17,19]. However, we performed an open splenectomy under the consideration of tumor size and difficulty mobilizing the spleen. Lobectomy for primary lung tumors is only guaranteed after successful splenectomy for the patient. Otherwise, the patient is inoperable, and systemic therapy or best supportive care will be given. We followed the patient for one year and there was no evidence of tumor recurrence or distant metastasis.

Table 1 lists the details of previous case reports of isolated splenic metastasis from lung cancer.

**Table 1:** Previous case reports of isolated splenic metastasis from lung cancer. HALS, hand-assisted laparoscopic surgery.

No.	Author	Histology (primary lesion)	Laterality	Time to splenic metastasis	Sex	Age	Metastasis symptoms	Treatment of primary lung tumor	Treatment of splenic metastasis
1	Reljic et al., 2022 [6]	Adenosquamous carcinoma	Left	144 months	M	56	Asymptomatic	Left upper lobectomy	Splenectomy
2	Kinoshita et al., 1995 [7]	Squamous cell carcinoma	Left	14 months	M	72	Asymptomatic	Surgical removal of primary tumor	Splenectomy
3	Massarweh et al., 2001 [11]	Poorly differentiated adenocarcinoma	Left	0 month	M	68	Splenic rupture	Palliative chemotherapy	Emergent splenectomy
4	Tanaka et al., 2020 [12]	Squamous cell carcinoma	Right	0 month	M	78	Splenic rupture	Surgery	Splenectomy
5	Lachachi et al., 2004 [13]	Poorly differentiated carcinoma	Right	0 month	M	77	Splenic rupture	N/A	Emergent splenectomy
6	Gupta et al., 1993 [14]	Squamous cell carcinoma	Right	0 month	N/A	N/A	Splenic rupture	N/A	Emergent splenectomy
7	Matsuoka et al., 2021 [17]	Adenocarcinoma	Right	0 month	F	69	Asymptomatic	Right middle lobectomy	Laparoscopic splenectomy
8	Nishikawa et al., 2017 [19]	Pulmonary typical carcinoid	Right	84 months	M	73	Asymptomatic	Right upper lobectomy	HALS splenectomy
9	Klein et al., 1987 [22]	Bronchioalveolar carcinoma	Right	20 months	F	57	Abdominal pain	Right lower and middle lobectomy	Splenectomy
10	Edelman et al., 1990 [23]	Poorly differentiated adenocarcinoma	Left	0 month	F	63	Asymptomatic	N/A	N/A
11	Macheers et al., 1992 [24]	Large-cell undifferentiated carcinoma	Left	0 month	N/A	N/A	Asymptomatic	N/A	Splenectomy
12	Takada et al., 1998 [25]	Bronchopulmonary carcinoid tumor	Right	96 months	M	49	Abdominal pain	Right upper lobectomy	Splenectomy
13	Tomaszewski et al., 2003 [26]	Lung cancer	Left	0 month	M	68	Asymptomatic	Left upper lobectomy	Splenectomy
14	Schmidt et al., 2004 [27]	Moderately differentiated adenocarcinoma	Left	25 months	M	72	Asymptomatic	Surgical removal of primary tumor	N/A
15	Pramesh et al., 2004 [28]	Squamous cell carcinoma	Left	2 months	M	55	Asymptomatic	Combined radiochemotherapy	Chemotherapy
16	Sánchez-Romor et al., 2006 [29]	Adenocarcinoma	Left	0 month	M	73	Abdominal pain	Left lung resection	Splenectomy
17	Van Hul et al., 2008 [30]	Adenocarcinoma	Left	24 months	M	67	Asymptomatic	Surgical removal of primary tumor	Splenectomy
18	Ando et al., 2009 [31]	Squamous cell carcinoma	Right	10 months	M	71	Asymptomatic	Combined radiochemotherapy	Splenectomy
19	Chloros et al., 2009 [32]	Squamous cell carcinoma	Right	0 month	M	59	Asymptomatic	Surgical removal of primary tumor	Splenectomy
20	Tang et al., 2010 [3]	Large-cell undifferentiated carcinoma	Right	4 months	F	49	Fever	Right lower and middle lobectomy	Splenectomy
21	Scintu et al., 1991 [33]	Large-cell carcinoma	N/A	0 month	N/A	N/A	Asymptomatic	Pulmonary lobectomy	Splenectomy
22	Yen et al., 2005 [34]	Adenocarcinoma	Left	24 months	M	56	Asymptomatic	Left pneumonectomy	Splenectomy
23	Fujii et al., 2008 [35]	Poorly differentiated adenocarcinoma	Left	3 months	M	58	Asymptomatic	Left upper lobectomy	Splenectomy
24	Assouline et al., 2006 [36]	Large-cell undifferentiated carcinoma	Right	21 months	M	77	Abdominal pain	Right pneumonectomy	Splenectomy
25	Eisa et al., 2014 [37]	Adenocarcinoma	Right	0 month	F	53	Abdominal pain	Surgical removal of primary tumor	Splenectomy
26	Belli et al., 2016 [38]	Large-cell carcinoma	Right	60 months	M	65	Asymptomatic	Right pneumonectomy	N/A
27	Sardenberg et al., 2013 [39]	Adenocarcinoma	Right	7 months	F	49	Abdominal pain	Right upper lobectomy	Splenectomy
28	Dias et al., 2012 [40]	Squamous cell carcinoma	Right	16 months	M	82	Asymptomatic	Right bilobectomy	Splenectomy
29	Cai et al., 2015 [41]	Adenocarcinoma	Right	17 months	F	56	Asymptomatic	Right lower lobectomy	Splenectomy
30	Soussan et al., 2011 [42]	Adenocarcinoma	N/A	0 month	M	52	Asymptomatic	N/A	N/A
31	Iguchi et al., 2015 [43]	Adenocarcinoma	Left	12 months	F	63	Asymptomatic	Left lower lobectomy	Splenectomy
32	Mitsimponas et al., 2017 [44]	Adenocarcinoma	Right	0 month	F	66	Asymptomatic	Radiochemotherapy	Chemotherapy
33	Hara et al., 2017 [45]	Poorly differentiated adenocarcinoma	Right	0 month	F	81	Asymptomatic	Right upper lobectomy	Laparoscopic splenectomy
34	Zeng et al., 2018 [46]	Adenoid cystic carcinoma	Right	48 months	F	38	Abdominal pain	Right middle lobectomy	Splenectomy
35	Lopera et al., 2018 [47]	Large cell carcinoma	Right	N/A	F	69	Abdominal pain	Right upper lobectomy	Laparoscopic splenectomy
36	Ousama et al., 2001 [48]	Non-small-cell lung cancer	Left	0 month	M	58	Abdominal pain	Chemotherapy	Splenectomy
37	Grant-Freemantle et al., 2020 [49]	Adenocarcinoma	Right	N/A	F	73	Asymptomatic	Right lower lobectomy	Splenectomy
38	Present case	Mixed adenocarcinoma and large cell neuroendocrine carcinoma	Right	0 month	M	68	Asymptomatic	Right upper lobectomy	Splenectomy

## Conclusions

There were limited cases reporting isolated splenic metastasis from lung cancer. Physiological and anatomical features can contribute to the rarity of splenic metastases. Isolated splenic mass is usually suggestive of primary lesion but care should be taken in patients with malignancies. There was no definitive treatment protocol in such a clinical scenario. Surgical treatment of isolated splenic metastasis may be extrapolated from those patients with oligometastatic adrenal or brain metastasis. Splenectomy is suitable to provide disease-free status and to prevent future splenic rupture if the patient presents a good performance status.
